# Disclosure in lesbian, gay and bisexual cancer care: towards a salutogenic healthcare environment

**DOI:** 10.1186/s12885-019-5895-7

**Published:** 2019-07-10

**Authors:** Julie Fish, Iain Williamson, Jayne Brown

**Affiliations:** 1 Centre for LGBTQ Research, De Montfort University, The Gateway, Leicester, LE1 9BH UK; 2 Division of Psychology, De Montfort University, Leicester, LE1 9BH UK; 3 Leicester Academy for the Study of Ageing (LASA) The Leicester School of Nursing and Midwifery, De Montfort University, The Gateway, Leicester, LE1 9BH UK

**Keywords:** Disclosure, Generalised resistance resources: healing environments, Salutogenesis, Sense of coherence, Sexual orientation, Qualitative methods

## Abstract

**Background:**

The literature on sexual orientation disclosure is arguably one of the most developed in the field of lesbian, gay and bisexual (LGB) people in healthcare in English speaking countries however, relatively little research has been conducted into disclosure in cancer care. Studies have been mainly undertaken in primary care where distinct circumstances pertain and where the benefits of disclosure include obtaining appropriate health information, treatment advice and avoiding misdiagnosis.

**Methods:**

We conducted an in-depth qualitative study primarily recruiting patients through oncology care in hospital settings and through LGB community cancer support groups. Data were gathered through semi-structured interviews with 30 LGB patients with different cancer types.

**Results:**

Data were analysed using thematic analysis and interpreted and interrogated through salutogenesis theory which offers a useful lens through which to consider the health promoting effects of sexual orientation disclosure in cancer care. We present three themes as part of the analysis: Authenticity as a driver for disclosure in cancer care, Partners as a (potential) salutogenic resource and Creating safe, healing environments conducive to disclosure. The findings are reported and discussed in relation to three inter-related concepts from current salutogenesis theorising including a sense of coherence, generalised resistance resources and healing environments which can facilitate sexual orientation disclosure.

**Conclusion:**

Our findings enable a more nuanced approach to understanding disclosure in this context. This study contributes to the literature through its articulation of the salutogenic potential of disclosure (if responded to appropriately) for LGB patients as individuals, in relationship to their partners or carers and the role of creating a visible healing-oriented optimal environment to promote quality of life and recovery.

**Electronic supplementary material:**

The online version of this article (10.1186/s12885-019-5895-7) contains supplementary material, which is available to authorized users.

## Background

The literature on sexual orientation disclosure is arguably one of the most developed in the field of lesbian, gay and bisexual (LGB) people in healthcare in English speaking countries. Yet despite its salience, there has been relatively little theorising of its potential benefits. Disclosure involves the communication of one’s sexual orientation to the clinician providing care. The significance of disclosure for the health and wellbeing of LGB patients lies in its association with improved psychological well-being [1]; however, findings about better physical health outcomes are more mixed [2]. Disclosure to health professionals brings multiple benefits including obtaining appropriate health information, treatment advice and avoiding misdiagnosis. Those who are open about their sexual orientation typically report greater levels of comfort with their healthcare providers and increased satisfaction with care [3].

Research in disclosure has been mainly conducted in primary care where distinct circumstances pertain; disclosure is often facilitated by the longevity of the relationship, it is usually one to one, and the interaction is typically patient initiated [4]. Moreover, LGB people often engage in a number of proactive strategies to identify a General Practitioner (GP) known to provide LGB affirmative care [5]. The timing of disclosure may also differ; in primary care disclosure is said to most often occur early in the relationship [6]. However, in cancer care, the circumstances may not be so conducive: the patient is in a potentially unknown environment, the consultation is often a formal and hierarchical one with the doctor leading the communication; moreover, at this early consultation, a patient may receive a diagnosis of a life-threatening condition.

Relatively little research about revealing a sexual minority identity has been undertaken in cancer care [7–9]. Recent research has used Meyer’s [10] concept of Minority Stress Theory (MST) as an explanatory framework to understand how expectations of rejection, experiences of discrimination and internalised homophobia may affect the likelihood of disclosure in cancer care [11]. The study highlighted both the effects of felt and enacted stigma and some of the strategies that LGB individuals use around the disclosure or performance of their sexual identity to avoid or deflect actual and/or anticipated conflict and hostility. In a systematic review, disclosure was also considered through the lens of minority stress theory: fear of discrimination, including receiving poor or unequal care, feeling embarrassment or humiliation after disclosure; concerns about confidentiality and the recording of sexual orientation in medical records were seen to constitute barriers to disclosure [4].

Taken together, these contextual factors suggest that the nature of disclosure in cancer care has a number of differing characteristics than in primary care and requires specific research enquiry. Findings may also go some way in explaining the lower rates of disclosure in hospital settings in comparison to those found in primary care [7]. In light of the above, the aim of this study is to explore the conditions under which a sample of British LGB cancer patients revealed their sexual orientation in hospital settings to enable a more nuanced approach to understanding disclosure in this context.

## Method

### Theoretical framework: disclosure as salutogenesis in LGB cancer care

Cancer has repercussions not only for people’s physical and psychological health, but also their identity and social well-being [12]. In approaches with a pathogenic orientation, a range of (typically unpleasant) medical treatments, including surgery, radiotherapy, hormone treatments and chemotherapy, may be used to remove signs of disease from the body. Quality of life and a resumption of activities that were the norm before illness, however, go beyond the absence of disease. Moreover, people’s health outcomes (including those specifically associated with cancer) are also affected by what Smith [13] has labelled ‘social pathogens’. These include factors such as health inequalities, stigma and prejudice, and poorly resourced healthcare that may lead members of certain social groups (such as the economically disadvantaged and members of black and minority ethnic communities as well as LGB individuals) to experience not only poorer health and well-being but also poorer quality, culturally insensitive care with its associated sequelae (lower satisfaction and participation in healthcare).

Whilst the current study sought to illustrate the vital role of such social pathogens in understanding British LGB people’s experiences of cancer care, our research also set out to recognise the resources that many LGB people utilise to improve their care pathway. It identifies some structural changes that can serve to synergise with these efforts, potentially leading to cancer care which is genuinely LGB affirmative. A key theoretical component here is ‘salutogenesis’, which as a health promoting orientation, underpins holistic care by addressing an individual’s social, psychological and personal factors, alongside the physical signs of illness [12], and identifies what Antonovsky [14] labels ‘salutary factors’ which can promote healing. At a simple level this may include identifying and nurturing both personal resources (such as resilience) and collective resources (such as social support from LGB friends and peers) and how these are employed and interact with the environment the individual is navigating.

Central to many understandings of salutogenesis is understanding how improved health may be generated by building on a person’s sense of coherence (SOC), which refers to how people view their lives, alongside their efficacy in utilising generalised resistance resources (GRR), which include ‘self-esteem, preventive health orientation, social support and cultural capital’ [15]. Movement towards the ‘health pole’ of the ‘ease/dis-ease continuum’ [14] is promoted by the presence of GRR.

Prior research has shown how LGB patients sometimes compartmentalise, or fail to disclose, their identities in healthcare settings [11]. For LGB patients with cancer, their SOC may include a holistic sense of self where they are able to be authentic in clinical interactions. Essentially coming out as LGB in cancer care illustrates a person’s self-acceptance and sense of coherence. In addition, taking pride or confidence in disclosure can be seen as a resistance resource. Integrating these multiple functions and meanings of disclosure provide a rationale for placing disclosure as a central process in understanding salutogenesis for LGB people receiving oncology (and potentially other forms of) healthcare.

Research in this field is flourishing with a growing literature on salutogenic approaches to health including work on older people [16], cancer patients [17] and HIV positive patients [18]. In addition, applied salutogenesis theorists have written extensively on how the policies, practices and structures of contemporary healthcare provision have the potential to create ‘optimal healing environments’ characterised by factors such as empathetic and compassionate care and cultural competence and sensitivity [19]. Therefore, both drawing on and contributing to this literature, this study considers the role of disclosure through the lens of salutogenesis theorising. In particular we consider the possibilities for promoting a sense of coherence, the potential role of partners in providing generalised resistance resources and salutogenic healing environments in LGB cancer care. The research questions which informed this study are:

What are the potential salutogenic factors that LGB cancer patients can draw on and how can this be enhanced in oncology care?

How is disclosure a potentially salutogenic resource for better coping with cancer for LGB people?

### Design

This qualitative study involved individual semi-structured interviews to elicit in-depth accounts of LGB people’s experiences of disclosure and nondisclosure, their interactions with professionals and their perceptions of good cancer care. The reporting for this study is informed by the 32 item COREQ criteria for qualitative health research [20].

### Public and patient involvement in the design of this study

Significant public and patient involvement (PPI) work was undertaken in developing the present research and to ensure it represents research *with* rather than *on* LGB people affected by cancer. PPI events were held in 2 British cities and open-ended questionnaires were distributed to participants at a LGB cancer inclusion event and at a structured discussion workshop at the research team’s university. In total 26 LGB people who have experienced cancer contributed views, 13 in a written form and 13 verbally, to the design and scope of the final study (Please see Additional file 1 for details).

### Participants

The inclusion criteria were that participants had been diagnosed with any form of cancer within the past five years and self-identified as a lesbian, gay man or bisexual person. Participants were recruited through non-probability methods, by displaying research posters which included a contact mobile number, in the clinic waiting rooms of five oncology departments in various parts of England and also through the online forum of a cancer charity, LGB social media and local radio interviews. Three potential participants withdrew from the study, due to a worsening of their condition, before data collection commenced.

### Procedure

Materials promoting the study were made available at participating hospitals in waiting rooms and through the websites of cancer charities and social media. Potentially interested participants contacted the research assistant and an initial discussion took place about the aims and purpose of the study. Participants who confirmed that they wished to take part were sent a participant information sheet, provided informed consent and gave brief demographic information. Interviews were guided by a semi-structured schedule which had been first piloted and was used flexibly (see Additional file 2: Table S1). Interviews lasted between 1 and 2 h and took place in a location of participants’ choice, mainly their own homes or in university or other private offices, and were conducted by one of four experienced interviewers, (three women and one man) all of whom had received good clinical practice training in 2017. No-one else was present for the duration of the interview. The interviews were audio- recorded and transcribed verbatim by a professional transcriber. Data were stored in a password protected file on a secure server.

### Ethics and research governance

A three stage procedure of ethical approval and research governance is required for research conducted in the NHS. Ethical approval was first obtained from the Principal Investigator’s academic institution, De Montfort University, Health and Life Sciences, Research Ethics Committee. Secondly, approval was awarded through the Integrated Research Application System to enable a study to be conducted in UK hospitals. Thirdly, NHS Research & Development, which monitors research governance procedures and requires a comprehensive audit trail for each hospital site was obtained. Ethical approval was conditional upon NHS training of all members of the research team. All data were securely stored and participants’ names and other details have been appropriately anonymised.

The study was conducted in accordance with the British Psychological Society (BPS), Code of Research Ethics [21]. Participants were provided with full information to enable them to take part and we obtained written informed consent. To ensure confidentiality and privacy, participants were allocated a pseudonym; a distress protocol was implemented and participants had the right to pause, reconvene or terminate the interview.

### Method of analysis

Our analytical approach is informed by thematic analysis [22]; this is a flexible method which is commonly used in health and psychological research. We paid attention to the meaning-making of participants in an iterative process. At phase one, at an early stage in the data-collection, four of the research team independently read a subset of transcripts. We met together to discuss our individual interpretations of the data and this immersive reading involved initial identification of patterns in the data. At phase two, two of the authors (JF and IW) developed a template to identify these recurring themes and produced an overarching framework. As the analysis developed we recognised that the concept of salutogenesis, defined by Jonas et al. [19] as ‘the process of healing and health creation’ provided a useful and relevant lens with which to interrogate and interpret the data at the latter stages of theme development and refinement. In this paper we present three themes: ‘*Authenticity as a driver for disclosure in cancer care*’, **‘***Partners as a (potential) salutogenic resource’* and *‘Creating safe, salutogenic healing environments conducive to disclosure***’**. The sequencing of our themes moves from individual through more relational and systemic influences on disclosure. Concepts from classic and current thinking around salutogenesis are applied throughout.

## Results

Thirty participants took part in the study (see Table 1). All participants identified as cis-gender with 18 men and 12 women participating. Of the men, 15 identified as gay and 3 as bisexual. Of the women 11 identified as lesbian and 1 as queer. Twenty participants were partnered and 6 were single. All relationships were with a same-sex partner apart from one man who was married to a woman and identified as bisexual. Participants’ ages ranged from 24 to 77 years. The cancers they had experienced were prostate [15], breast [9], lymphoma [2], thyroid [1], ovarian [1], bowel [1] and skin [1].

**Table 1:** Participants’ demographic characteristics

P #	Pseudonym	Identity	Relationship status	Age	Cancer site
1	Fiona	Lesbian	partnered	55–64	Breast
2	Julian	Gay man	partnered	55–64	Prostate
3	Rudy	Gay man	partnered	65+	Breast
4	Bob	Gay man	partnered	65+	Prostate
5	Nadia	Lesbian	partnered	35–44	Breast
6	Tom	Gay man	partnered	45–54	Prostate
7	Robert	Bisexual man	partnered (with a man)	45–54	Prostate
8	Daniel	Bisexual man	married (with a woman)	55–64	Prostate
9	Jeremy	Gay man	partnered	65+	Prostate
10	Steph	Queer	partnered	25–34	Thyroid
11	Corinne	Lesbian	partnered	55–64	Breast
12	Ellie	Lesbian	single	65+	Breast
13	Lou	Lesbian	partnered	24–34	Breast
14	Robin	Gay man	partnered	45–54	Prostate
15	Terry	Gay man	single	65+	Prostate
16	Quentin	Gay man	partnered	55–64	Prostate
17	Miranda	Lesbian	partnered	35–44	Breast
18	Linda	Lesbian	partnered	45–54	Breast
19	Tracy	Lesbian	partnered	45–54	Bowel
20	Davina	Lesbian	partnered	55–64	Ovarian
21	Karl	Gay man	partnered	55–64	Prostate
22	Tessa	Lesbian	single	45–64	Breast
23	Oliver	Gay man	partnered	35–44	Lymphoma HIV+
24	Gertrude	Lesbian	Single	65+	Lymphoma
25	Noel	Gay man	Newly partnered	65+	Prostate
26	Tim	Bisexual man	Partnered (male)	65+	Prostate
27	Nigel	Gay man	Single	35–44	Prostate
28	Liam	Gay man	Partnered	35–44	Skin
29	Craig	Gay man	Partnered	65+	Prostate
30	Norman	Gay man	Single	55–64	Prostate

### Authenticity as a driver for disclosure in cancer care

Normative assumptions underpinning healthcare are that the patient is heterosexual - their identity/ sexuality does not require articulation - in most interactions it is simply and implicitly presumed [23]. For LGB patients, authenticity is achieved by a positive response to the disclosure of sexual orientation and a shared recognition by both patient and professional that the whole self is relevant to health, rather than a focus on merely treating the disease. However, a minority of participants chose not to disclose their sexual orientation to health professionals arguing that it was not related to their care:

I didn’t feel any need to tell them that I am gay because it was in my view irrelevant to treatment. But they certainly didn’t ask me and why would they? (Bob, gay man, prostate).

Here, Bob, employs a pathogenic orientation to his cancer care. He implies that his non-disclosure is an active choice that allows all parties to concentrate on the biomedical ‘business’ of treatment and recovery without ‘irrelevant’ distractions. This construction is further reinforced by his rhetorical question.

However, other participants said that coming out to health professionals early in the relationship enabled them to decide how and when to disclose. By choosing the timing or circumstances of disclosure, Noel said that it took the stress away from a subsequent moment where he may have felt more vulnerable. Moreover, for him, disclosure enabled ordinary conversations which facilitated everyday social interaction:

almost invariably…you talk just for filling time about your family life, …And if you’re just holding that just one bit of information back it gives a little bit of stress. …. I made the decision at the outset, I’d said that I was gay (Noel, gay man, prostate).

Relating to salutogenesis, taking control of disclosure connects to the key concept of sense of coherence wherein Noel uses voluntary and self-initiated disclosure to manage both current and future potentially stress-inducing situations (Eriksson, 2017). Others voiced more empathetic motives for disclosure arguing that they facilitated authentic relationships with others:

It wasn’t a case of us being asked… we volunteered the information because it makes other people feel at ease (Davina, lesbian, ovarian).

Other participants said that disclosure facilitated holistic care; Rudy clearly identified the benefits of being open with health professionals, saying that being gay is the first thing that they should know about him:

As far as I’m concerned that unless you are open about your sexuality you can’t expect to be treated holistically… If I am on my own I make sure that people know. I think it’s important for other people…who might not be so confident (Rudy, gay man, breast).

In contrast to participants like Bob, Rudy articulates a perspective that disclosure is fundamental to holistic or person centred care. Such an approach, in salutogenesis theorising, might be understood as ‘facilitating healing’ rather than simply ‘being treated’ [19]. He also appears to take pride as a role model for other less self-assured LGB people.

Many participants addressed the potential presumption of heterosexuality in initial interactions with cancer specialists, recognising that staying ‘closeted’ would both engender feelings of anomie and deny them access to full psychosocial care:

I did say very early on that I was gay and I didn’t want people to assume that I was straight…because it makes me feel alienated. They are not actually engaging with the real me (Terry, gay man, prostate).

Because of the prevalence of prostate cancer amongst our gay and bisexual participants, our male participants were mostly over the age of 55. Tim reflected that gay and bisexual men who came of age at the time of the 1967 Sexual Offences Act represented a generation that is ‘not comfortable in own its skin’ and hold deeply ingrained attitudes ‘that you can’t be yourself’. However, in choosing to disclose, he believes he has received superior care:

I have been able to have a smoother journey through this because I didn’t keep anything hidden (Tim, bisexual man, prostate).

Whilst taking active control of disclosure early in their cancer pathway was the most common narrative, a small number of participants discussed how their cancer diagnosis represented a catalyst to a more open and ‘authentic’ way of managing their sexual identity. For Robert, who identified as bisexual, a supportive new relationship with a male partner, combined with therapeutic support and led to a watershed moment:

I am completely open now about my sexuality which I wasn’t before when you have only got a certain amount of time…what is important is being honest and authentic (Robert, bisexual man, prostate).

As acknowledged above, a minority of participants saw their sexual orientation as irrelevant to their cancer care. They talked of not wanting the ‘label’ of sexual orientation or of feeling that they have received or should receive the same quality of care as do other patients without a need to voice being lesbian, gay or bisexual. Other participants argued, however, that openness about their sexual orientation meant that they received appropriate psychosexual advice for side-effects of prostate cancer such as erectile dysfunction while others were motivated to disclose because it creates a smoother or more humane relationship. These participants said that disclosure enabled professionals to engage with them in an holistic and authentic fashion and gain the whole picture of their lives. Disclosure also provided a sense of coherence and self-efficacy which facilitated their navigation of their cancer journeys.

### Partners as a (potential) salutogenic resource

Having a partner or carer, and particularly one who attends medical appointments, makes it easier for participants to disclose (and thereby gain validation for the relationship). In some cases explaining their presence obliges the patient to disclose their sexual orientation. Here, we explore how partners may comprise a salutogenic resource if their supporting role is recognised and encouraged; conversely, their role may be invalidated if overlooked, or dismissed by healthcare professionals.

Four-fifths of participants in our sample were in a relationship, and all (but one of them) were accompanied by their partner to hospital consultations and treatment. Although Robin had not explicitly disclosed his sexual orientation, the healthcare professionals involved in his care implicitly validated their relationship:

we haven’t just come out and said we are gay, we are married but …the way they talk, oh [you] are together. And they don’t change their attitude, it does make you feel at ease and it does help you with the course of action. They appreciate he is here for me (Robin, gay man, prostate).

He draws attention to the benefit of his partner’s involvement in making decisions about treatment options or ‘course of action’. In a similar vein, Steph recognises that disclosure enables health professionals to ‘treat me as a whole person’ within an ecological framework including ‘family, support network and partner’:

People don’t get the full picture of your life otherwise and who is supporting you (Steph, queer, thyroid).

A partner’s presence also provides the opportunity to disclose without having to make a direct statement about oneself, when there is no other context in which to place such a statement. This strategy of active disclosure links to the forthright approach adopted by many participants in theme one and to the concepts of authenticity and sense of coherence. A number of participants talked about staff being ‘fine with it’, ‘not uncomfortable or uneasy’. Others talked about interactions with staff where they felt, rather than a fairly passive acknowledgement, that the nurse had actively validated their relationship, in this instance by a positive comparison with her own:

(she)…pushed me down to the theatre and she went “have you been together long?” and I said oh 22 years… and she went “good lord that’s longer than me and my husband- you deserve a medal”. So, perfectly accepting and perfectly friendly. (Liam, gay man, skin).

Nadia talked about an interaction which illustrates (tacit) knowledge of generalised resistance resources [24] wherein the breast care nurse actively extends her (Nadia’s) coping resources, promoting her social support and paying attention to what is working well:

My marvellous breast care nurse …said you get your wife to make you an Ovaltine in the evening, get her to bring it up to you in bed. And it was the way that she so naturally said that, it was absolutely lovely, it was moving because we never really had an explicit conversation about that (Nadia, lesbian, breast).

Reflecting on the nurses’ involvement of his partner in planning his care, Karl describes a proactive approach in which his partner is recognised as a resource to promote his health, in addition to showing concern for his partner’s own health as a carer:

they ask him how he is…they talk to him about him as well (Karl, gay man, prostate).

In contrast to these salutary/health-promoting experiences, participants talked of interactions in which their significant relationship was invalidated (and the potential was missed for a salutogenic approach): partners were assumed to be siblings, parents or friends. In some instances, the couple had attended appointments together sitting in a waiting room over several weeks and were ignored, while they perceived that heterosexual couples in a similar situation were engaged with. Sometimes a professional would make assumptions, or ignore information rather than ask an open question for clarification. The partners of two female participants were metaphorically shut out by a curtain during a biopsy because the health professional told them they were just ‘good friends’, while a gay man’s partner was whispered about as if he were a secret.

A number of participants talked about the legal recognition afforded to their relationship by the Civil Partnership Act 2004 and/or the Marriage (Same-Sex Couples) Act 2013. They claimed legitimacy for their relationships by introducing their partner as their husband or wife, but this was disputed by some health professionals. For example, one woman said:

I was admitted there was a form (and) the woman …argued that we must be civil partners that we couldn’t be married. (It) went on for slightly longer than was comfortable. Presumably she hadn’t heard…it had changed now ….. That somehow …we were mistaken that we were married (Lou, lesbian, breast).

Often, reflecting on the interactions they had, they compared the treatment of their partner with what they saw offered to others, where heterosexual couples did appear to be involved. But at other times, they did not know whether they were treated differently to other couples.

In cancer care, patients are commonly accompanied by their spouse and can often help to process information and support them emotionally if they should receive bad news. However, in the following instance, the doctor’s discomfort meant that she ignored Jeremy’s partner in the consultation:

..the one consultant at Anycity hospital was uncomfortable …I felt as though she didn’t know how to deal with a gay couple. She was uncomfortable with us …she wouldn’t talk to us, she would sort of talk to me, but Barry …might as well not have been in the room…her manner was cold, and I felt the way that she delivered what was virtually a death sentence (Jeremy, gay man, prostate).

In these latter experiences, participants were denied complete access to the salutogenic resource embodied by their partner or carer. If the partner’s role is not acknowledged, they may be less likely to play a part in making sense of a diagnosis or comprehending treatment plans. By contrast, experiences of validation draw on salutary factors in cancer care, most notably the desired outcomes of ‘reassurance of worth’ (as an LGB person with cancer), providing opportunities for ‘nurturing’ and laying the foundations for a ‘reliable alliance’ with their carer in promoting recovery and quality of life [25]. There were, however, several instances where partners were validated by health professionals and the relationship was quietly accepted or openly acknowledged.

### Creating safe, salutogenic healing environments conducive to disclosure

Recent work on salutogenesis has considered how elements of both the interpersonal and material environment of hospitals can profoundly shape the extent to which cancer care is delivered in what have been described as ‘optimal healing environments’ (Jonas et al., 2014). Many of our participants in the study scanned the environment for visible clues about the hospital ethos, looking for signs of its commitment to equality and diversity:

I want to recover and I don’t want the issues [of homophobic prejudice]…Maybe I am the only one who thinks that having a little rainbow flag matters, but it does, and you think oh great, I am not going to have to give a thought about this (Corinne, lesbian, breast).

The presence of a sign or symbol has the potential to act as a visual reassurance of a salutogenic environment for participants; Corinne, said she would be assured that she would not encounter poor reactions from staff meaning that she could then focus on her recovery. However, the hospital appeared not to display any materials to indicate its policy on inclusivity, despite it being a major cancer centre serving a city noted for its large LGB community. For another participant, the lack of a visible healing-oriented optimal environment meant that he went back into the closet:

I was in this strange alien environment is how I viewed it, there was nothing there to reassure me that I would be OK being myself. I am out in every other aspect of my life I went back in the closet for this (Julian, gay man, prostate).

By contrast, another participant pointed to a symbol worn by a member of staff as evidence of an optimal healing environment:

Whilst I was in hospital there was a girl there who had a rainbow tie…it would have been nice…to have had more things around that were identifiably LGBT. (Ellie, lesbian, breast).

Ellie was reassured because one member of staff felt sufficiently comfortable to wear the rainbow symbol. Through this she inferred acceptance from the wider team. Markers of an LGB presence and especially a visually identifiable commitment to rights, however small, were very important on participants’ sense of ease. The lack of such symbols, reported in almost all hospital contexts, had the potential to make participants feel not fully safe. For some it influenced their nondisclosure thus denying them access to full psychosocial support.

Alongside a lack of visible signs of equality indicators, some participants overheard casual conversations including homophobic discourse amongst staff which had an impact on perceived levels of safety:

And I’d had a few… they weren’t directed at me but throwaway remarks ….just generally about gay blah blah blah (Julian, gay man, prostate).

Several participants experienced greater discomfort in public environments, such as waiting rooms or hospital wards, which involved contact with other patients. They described the atmosphere as ‘a bit of awkwardness’ or ‘you don’t quite fit with the normal things that they say and do’ or they often chose to close down conversations about family relationships and children. In particular, several of the gay and bisexual men in the study described feeling vulnerable and threatened by the atmosphere on all-male wards:

And so they put me in the orthopaedic ward and I felt really, really threatened by being with a whole load of extremely macho men… I didn’t like that at all and so I went back into the closet straight away. And so it led to things like, I said to Matthew, (his partner) don’t kiss me. And …I even encouraged him…you needn’t bother to come and visit (Rudy, gay man, breast).

Although Rudy’s concerns relate to an anticipated rather than enacted prejudice, his sense of threat from other patients undermined his earlier strongly articulated commitment to disclosure. For him, the wider hospital environment meant that he was not able to benefit from visits by his partner. Thus, he elects to distance himself from a key salutogenic resource (the social support provided by regular visits from his long-term partner) at a time when he is both physically and psychologically at his most vulnerable. Other participants also commented on how visiting times on a hospital ward were occasions where sexual orientation became visible. Ellie describes her discomfort about being visited by lesbian couples:

I don’t think I did (disclose) particularly. I think the thing is the people that were visiting me …. they come in couples ...so I think anybody who was looking after me would know that I was a lesbian (Ellie, lesbian, breast).

Public displays of affection (such as hugging or kissing) were constrained by the presence of other patients; gestures of affection were typically minimised (especially amongst male participants) while other participants noted that their affection was not expected by other patients:

when you come into hospital and they (your partner) give (s) you a peck on the lips, and I think that’s an interesting thing for other people in the ward. Because maybe they are not expecting it (Corinne, lesbian, breast).

For Lou, being in a same-sex relationship meant that her partner was able to have additional access to spending time with her on a single sex ward when male partners were not allowed. Nevertheless, she describes constraining their behaviour ‘so we wouldn’t have caused any noise or trouble’.

Whilst many participants discussed feeling uncomfortable under a heterosexist ‘gaze’ of fellow patients, some participants disclosed positive experiences where their partner and their children were acknowledged by other patients.

Participants were unable to recall any visible LGB-affirmative symbols in the hospitals where they received treatment although smaller markers of inclusivity were occasionally signalled by individual health professionals. For some participants, low key acceptance provided assurance that they would not encounter prejudicial attitudes, while others preferred a brief, positive acknowledgement of their identities. Although no participant expressed concern about their medical treatment, generally the environments described by participants could not be described as ‘optimal healing environments’.

## Discussion

The study makes a contribution to the emerging body of literature on sexual orientation disclosure in cancer care [26, 27]. While the literature has focussed on the need for disclosure in countering the presumption of heterosexuality [28], this study articulates the salutogenic potential of disclosure for LGB patients in enhancing quality of life and recovery. Below we revisit each of our themes and consider their relevance of our findings for a nuanced approach to understanding disclosure in secondary care.

### Authenticity in cancer care

Previous studies have suggested that many LGB patients only disclose their sexual orientation if they perceive relevance for the treatment of their cancer; moreover, this is more likely if their cancer is related to sexual or gynaecological health [5]. Yet, a recent study in Scotland found that 40% of men who have sex with men believed that sexual orientation was not relevant in primary care [29]. While sexual behaviour and health are significant in the lives of LGB people, many construct their identities in a multi-dimensional way including: identity (perceptions of self), desire (who one is attracted to and preferred sexual behaviours) and community (social support and connectedness to others) [30]. The association of disclosure with sexual health to the exclusion of other dimensions of identity may result in a narrowly defined conception of LGB people’s sense of self. Authenticity in cancer care may only be possible if patients are supported in incorporating their multifaceted complexities rather than a reductionist view that posits LGB identities as solely located in sexual behaviours. Being authentic then is a foundation for the giving and receiving of holistic, inclusive, person-centred or comprehensive care which enables the health professional to take account of the physical, emotional, psychological and social needs of the individual and the impact of cancer on their capacity for self-care to promote well-being and recovery [31]. Our interpretation of the findings in this study are that sexual orientation is always relevant and is fundamental to achieving authenticity, but only if disclosure is responded to positively. The UK Department of Health’s flagship assessment of LGB patient experience found differences on 24 measures including disagreement with the statement ‘I never felt treated like a set of symptoms rather than a whole person’ [32]. If a patient feels that their cancer is treated without regard for them as a whole person, they may feel that the cancer professional is solely concerned with treating their disease or has not considered that treatment options might affect them differently [33]. These data are confirmed in a study of cancer patients’ priorities, wherein participants rated the management of practical, social and emotional issues as a higher priority than biological and treatment aspects [34].

### The partner as a potential salutogenic resource

In the wider literature, partners can play a key role in gathering information and form part of the triad in the consultation process [35]. This study, which evaluated the degree to which opposite sex partners of prostate cancer patients participated in shared decision-making, showed that the doctor encouraged the spouse to participate and found, among several benefits, strong associations between satisfaction with treatment options and the active participation of the partner or carer.

Our findings that the presence of a partner can act as a prompt to sexual orientation disclosure to a professional confirms other findings in the literature [36] and can thus promote self-actualisation and authenticity. But the distinctive contribution of the findings lies in participants’ recognition that validation of their relationship helps them to feel at ease and accepted. Moreover, the findings point to generalised resistance resources such as helping to make treatment decisions and greater awareness of the social support a partner may provide.

### Creating safe, salutogenic healing environments conducive to disclosure

The presence of visible symbols to signal that a hospital is dedicated to providing comprehensive care was perceived to be lacking by participants in this study. There were circumstances where individual health professionals wore a brooch or a tie, but there were no examples of a whole system approach to LGB inclusion. Public areas within hospital settings appeared to present some tensions such as in waiting rooms and on hospital wards where participants were concerned about reactions from other patients. In these environments, they were constrained by feeling unable to seek reassurance by a hug or a kiss. These findings support prior research which has shown a lack of culturally competent care reflected in patient intake forms, provider-patient communication and confidentiality [37]. While several participants were prepared to discuss the impact of treatment for prostate cancer for their future sexual relationships, some participants felt that this was an additional responsibility which they did not want to undertake confirming findings elsewhere [38]. There were few examples of homophobic remarks, but they created an alien environment. LGB affirmative care symbols, then, represent more than a token gesture and would indicate to LGB patients that staff were trained and committed to providing an inclusive service [39]. It would also mean that health professionals were aware of current marriage legislation so that the status of a relationship is not contested. These issues problematise the notion of comprehensive care; in common understandings, equality in care is taken to mean: ‘I provide the same care for everyone’, the objective for quality healthcare is equity and not sameness [40, 41]. The findings illustrate some of the complexities of facilitating disclosure. Some of our study participants expressed the preference for staff to be openly LGB affirmative, but they did not always want other patients to know. This is something of a paradox in the creation of LGB clinical friendly spaces.

### Limitations

This study has a number of limitations which may be reflected in the sample: there is some evidence to suggest the majority of LGB people live alone [42], but in our sample four-fifths were partnered. We were unable to recruit LGB people from Black and Minority Ethnic communities or bisexual women. Participants were recruited through hospital trusts which had already provided LGB equality and diversity training for staff and who might be expected to be more likely to take inclusive approaches to care. Participants were also recruited through cancer support groups and were therefore more likely to be open about their sexual orientation and to consider it relevant to their care. The study is not intended to represent the experiences of all LGB patients with cancer, but to present detailed first-hand accounts about how participants manage disclosure to health professionals, their perceptions about the relevance and value of disclosure to their sense of self and their experience of care and their responses to the hospital as a potentially healing environment.

## Conclusion

In conclusion, this study illustrates three domains with health generating potential for LGB patients with cancer: promoting authenticity in healthcare interactions; involving a partner or carer in the cancer care pathway and the creation of healing environments. Taken together, the three domains constitute a salutogenic orientation for LGB cancer care. Such an orientation of necessity asks: ‘how can this person be helped to move toward greater health in all aspects of their humanity?’ Recognition of this principle would enable professionals to facilitate disclosure or to create optimal healing environments which would benefit those patients who did not see the need. Creating the conditions which signal inclusivity and allow for the possibility of disclosure contribute to moving LGB people to greater health, whether or not they are able to disclose. Further research which asks professionals about strategies to achieve these outcomes is needed.

### Recommendations

The study findings first highlight the need for training of all staff where they are able to facilitate disclosure, make a positive response and promote understanding of its health benefits. Rather than the individual markers of inclusivity noted by participants, the study recommends a whole system approach across secondary care in the NHS. In the USA, the healthcare equality index confers an award to healthcare organisations which acts a kite-mark of quality assurance. In the UK, localised projects have introduced similar initiatives in primary care, but no England-wide initiative currently exists to support this work in hospital settings. Second, the recognition of partners or carers as a salutogenic resource in the care pathway can contribute to promoting quality of life. Third, the creation of LGB healing environments where visible symbols of inclusion act as quality assurance mechanisms that staff are knowledgeable about LGB cancer care and have the skills and confidence to support health promoting outcomes. In these environments, health organisations will have worked to eradicate homophobic discourse and develop settings where LGB patients are fully comfortable in receiving visits from family and friends. These are all key processes that need to be developed in ensuring that LGB people feel fully satisfied with their care.

## Additional files

Additional file 1: Including PPI in study design of LGB cancer care (DOCX 23 kb)

Additional file 2: **Table S1.** Promoting good outcomes in Lesbian, Gay and Bisexual (LGB) cancer care: a qualitative study of patients’ experiences and interactions with health professionals in clinical oncology (DOCX 105 kb)

Additional file 3: Supplementary Information relating to the Hospital Sites (DOCX 12 kb)

## Data Availability

The datasets used during the current study are available from the corresponding author on reasonable request.

## Abbreviations

GRR: Generalised Resistance Resources
LGB: Lesbian, gay and bisexual
MST: Minority Stress Theory
PPI: Public and patient involvement
SOC: Sense of coherence
