# Supplementary material for: Disclosure in lesbian, gay and bisexual cancer care: towards a salutogenic healthcare environment
Source: BMC Cancer. 2019 Jul 10;19:678. doi: 10.1186/s12885-019-5895-7 (PMC6617610; doi:10.1186/s12885-019-5895-7)
Supplement: Supplementary file 1 — Including PPI in study design of LGB cancer care (DOCX 23 kb) [file 12885_2019_5895_MOESM1_ESM.docx]

**Including PPI in study design of LGB cancer care**

***Recruitment of cancer patients to give their views on the study design***

A cancer support charity held a workshop in a Northern city for LGB cancer patients to elicit their views about appropriate support and relevant information. The survey (five questions) was distributed to participants at this event and 13 completed questionnaires were subsequently received. Two months later we held a workshop for LGB cancer patients and asked them to reflect on the survey responses. These are collected below.

Survey responses (13 participants)

| P # | Q1 **What do you think are the most important issues** | **Q2 Are there any questions this proposed study should ask** | **Q3 what output or resource would be useful for patients** | **Q4 what output or resource would be useful for professionals** | **Q5 anything you’d like to say here** |
| --- | --- | --- | --- | --- | --- |
| 1 | Equal treatment not the same treatment | Ask me what I think should have happened in the first few months I was engulfed in the system compared to what actually happened | Mainstream patient info to include sections for LGBT patients | All training for all roles/positions to include fully comprehensive LGBT awareness backed up with specific training for different cancer specialties | I was a bit shocked yesterday to find this comment in a NICE publication  Clinical Knowledge Summaries, Erectile Dysfunction, prescribing information: Tadalafil 5 mg taken once daily can improve overall satisfaction for men and their female partners. |
| 2 | Advice and info tailored to me a gay man not a straight man |  | Separate LGBT focussed info for patients | Initial LGBT training to be a first step not the last one | Understanding without judgement |
| 3 | A welcoming and supportive environment possibly with poster etc to state that all LGBT patients are welcome and will be supported |  | Booklet website explaining how different cancers can affect LGBT people differently | Information about the special concerns for LGBT patients | Look at study being conducted by kings’ college basically the same type study |

| 4 | Fair and honest treatment | Nothing specific, although it might be useful to ask about negative experiences with medical teams | LGBT support groups | Networks | I would like to be involved in something that creates a multi faceted LGBT peer support system |
| --- | --- | --- | --- | --- | --- |
| 5 | Being treated as an individual and acceptance of self partner carer family | Need to train | Information | What could be unique about this cancer treatment because person is LGBT |  |
| 6 | Services access treatment acceptance | Feelings in waiting area / doctors and professionals | Cultural and LGBT specific | Training |  |
| 7 | For sexual identity to be acknowledged and addressed in the way the patient needs |  | There is specific advise for gay men from Prostate Cancer UK but it would be good if there was specific advice for other LGBT cancer patients Support groups | Information on LGBT community/ Access to info at all levels | I work in research & evaluation within palliative care and would be interested in learning more about this study |
| 8 | Acceptance, being treated as individuals tailored information |  | Specific literature and peer support | A module of training addressing LGBT issues | It’s good to see positive movement/ Marvellous personnel – friendly courteous |
| 9 | Knowledge & understanding | How can we improve services for LGBT cancer patients | A closed forum – not openly accessed by ‘sightseers’ | Education at undergrad and professional level |  |
| 10 | Emotional and psychological support |  | LGBT specific info leaflets supported by improved training for professionals | A CD or DVD – on various issues |  |
| 11 | Recognition of partners and possibly family dynamics |  | A CD or DVD – on various issues | A reference document | Our NHS is great but if my experience us typical it has a lot to learn about LGBT people |
| 12 | Being ordinary in a knowledgeable way | What does an LGBT-affirmative clinical environment look like? | A reference document | Specific needs (physical and psychological) of men women LGBT etc |  |

**Comments/discussion in the workshops (13 participants) summarised by the facilitators:**

13 I am a gay man and was diagnosed with prostate cancer in December. I told my MacMillan nurse about my sexuality and she told the oncologist and surgeon who saw me at the first meeting to discuss treatment. The surgeon didn't seem to tailor what he said to me because of my sexuality and I even had to ask him a question to get him to confirm that after a prostatectomy I wouldn't ejaculate. I think the most important issues for LGBT patients is that the medical should be trained so that they have some understanding of the different issues facing LGBT patients compared with Heterosexual patients. When I was about to be discharged from hospital after HDR Brachytherapy I asked the junior doctor when could resume sexual intercourse. She seemed a bit embarrassed and simply said it would be okay when I felt like it. Of course I meant anal sex and I am often the receptive partner. She made an assumption and didn't ask about my sexuality. Given her answer I didn't bother explaining and did some research on-line for advice.

14 Many participants at the workshop were struck by the importance of ‘micro-interactions’ or ‘micro-moments of opportunity’ when patient-HCP relations could be influenced. They felt this resonated with their experiences and would be good to capture in future research in more detail .

15 Developing LGBT affirmative care – what should it look like? Sharing excellent practice.

16 Depression and anxiety

17 How pain is managed

18 Issues around fertility and reproduction

19 How do LGBT manage disclosure on the ward – i.e. to other patients? What role do HCPs feel they have in creating an LGBT-affirmative ‘social’ environment.

20 Capturing stories of resilience and survival. Thinking about the assets and resources that many LGBT have

21 What have been the nature of good experiences? How do we build on that and make those more common.

22 There were some concerns about when and how to engage participants in research. Many favoured collecting only retrospective accounts but others felt that more imaginative methods (dictated or written diaries, photos, drawings (including ‘drawing cancer’, use of apps) could facilitate data closer to the experience of illness and treatment. It felt

23 Some contributors felt that participants should be offered a choice in how they take part (i.e. in person or over the phone/internet; focus group or individual interview)

24 Some felt it was important that people could take part in a very discrete or private way if they wished.

25 There were some discussions about how participants could/should be ‘grouped’ to ensure reasonable representativeness of cancers as well as LGB(T) identities.

26 This was sent by email:

This is a great project. Please don't be put off by my feedback....

Please see comments on the patient interview and the research proposal (attached as word files with comments.)

I haven't commented on the focus group schedule in detail because I have big reservations about the use of this method for your study.  I think the questions are likely to be answered with dull platitudes like - We treat everyone the same, I don't have a problem with anyone's sexuality...blahh, blahhh - dull and not very useful for improving services;having colleagues in the same room would only add to the defensiveness.  Better in my experience to do this in an interview where the researcher can give plausible assurances about confidentiality. As well as the general questions about LGBT care, I would also ask them to focus on the last person they cared for who was LGBT. This method has for me yielded more useful info than more general questions.  Vignettes might also be a useful way of drawing out attitudes from people who do not think its an issue.

I have a general comment about both of the schedules which is about their pace and order.  This is probably only relevant if you get the funding but, if you do, it would be good to think about the order of the questions within each of the sections so that there isn't too long a run of the more challenging/sensitive questions.

| Taking account of your feedback we did… | The overall research approach | Methods | Interview schedule for patients | Outputs- engagement -influencing the delivery of cancer care |
| --- | --- | --- | --- | --- |
|  | We were attentive to the notion for sexual identity to be addressed in the way the patient needs. We discussed this in our project steering group meetings and agreed that we would not imply that disclosing sexual identity was the only response to make. | We designed vignettes for the focus groups with cancer professionals but did not receive funding for this part of the study | We reduced the number of questions in the interview schedule and reframed the Qs as domains | We held a public engagement event to consider the views expressed in the survey with workshop participants |
|  | We decided against asking a Q about negative experiences – as we believed that the term ‘experiences’ would encompass positive **and** negative ones |  |  | We have published the findings in a professional journal |
|  |  |  |  | We produced a final report that was widely available in print and digital formats |
|  |  |  |  | We have contributed to an online multi-faceted peer support system |
