# Supplementary material for: Disclosure in lesbian, gay and bisexual cancer care: towards a salutogenic healthcare environment
Source: BMC Cancer. 2019 Jul 10;19:678. doi: 10.1186/s12885-019-5895-7 (PMC6617610; doi:10.1186/s12885-019-5895-7)
Supplement: Supplementary file 2 — Table S1. Promoting good outcomes in Lesbian, Gay and Bisexual (LGB) cancer care: a qualitative study of patients’ experiences and interactions with health professionals in clinical oncology (DOCX 105 kb) [file 12885_2019_5895_MOESM2_ESM.docx]

**Table S1: Promoting good outcomes in Lesbian, Gay and Bisexual (LGB) cancer care: a qualitative study of patients’ experiences and interactions with health professionals in clinical oncology.**

**Interview topic schedule for patients**

As you know, this interview is part of a research project which is looking specifically at the experiences of lesbian, gay and bisexual people who have received a cancer diagnosis. I’m here to interview you about your experiences and feelings in relation to your journey through your cancer care and treatment and being LGB.

I would just like to reassure you that all the information you provide me with will be kept confidential, and will be anonymised so that you won’t be identifiable from the research findings.

If at any time there is a question you don’t feel comfortable with, or if you want me to stop the interview, just say so and I will stop immediately.

**Opening question:**

Can you tell me about the events leading up to your diagnosis and first receiving the diagnosis from the hospital doctor?

**Decisions about whether to disclose to a cancer professional:**

- Did you disclose your sexual orientation or gender identity at your first appointment?
- Had you been intending to disclose?
- What made you decide to disclose?
- What reaction did you get?

**Involving carers or partners in treatment decisions**

- Did you have a stay in hospital?
- Was your partner/carer involved in deciding best treatment for you?
- How easy or difficult was it for your partner/carer to be involved?
- How was your relationship with your cancer care professionals?
- How was their relationship with your partner/carer?
- Are there any particular experiences of care you want to talk about? (Operation, radiotherapy, chemotherapy, management of pain)

**Accessing Information**

- How easy or difficult was it to access information and sources of support for LGBT people?
- Did you feel this was helpful?
- Were staff informative or knowledgable?

**Social Support**

- Who do you talk to about cancer?
- What kinds of informal support have you had? (friends, partner, ex-partner(s), family, neighbours, work colleagues?).
- Who do you get the most support from?
- What kinds of support do they/have they given?
- Did your support system ‘fit together’?

**Recognition of your support and care needs and what you think good care involves**

- How would you describe your quality of life?
- Do you think you have particular support needs being a LGB person?
- How would you describe the care you have received/are receiving – good /bad /ok /could be improved, etc.
- What are your feelings and attitudes towards cancer?

**Finally**

Is there something you would like to say that I haven’t asked about?

Is there something you would like to ask me?

**What will happen to the information you have given me.**

We will anonymise the information-in other words your name and any information which would identify you would be removed. We will then put all the information together and draw out themes, if people have said similar things. We will then write a report which will describe the experiences LGBT people with cancer have in relation to the care and treatment which is proved for them.
