# Supplementary material for: Disclosure in lesbian, gay and bisexual cancer care: towards a salutogenic healthcare environment
Source: BMC Cancer. 2019 Jul 10;19:678. doi: 10.1186/s12885-019-5895-7 (PMC6617610; doi:10.1186/s12885-019-5895-7)
Supplement: Supplementary file 3 — Supplementary Information relating to the Hospital Sites (DOCX 12 kb) [file 12885_2019_5895_MOESM3_ESM.docx]

Supplementary Information relating to the Hospital Sites

The Integrated Research Application System (IRAS) number is 180271. The participating hospital sites were: Clatterbridge Cancer Centre NHS Foundation: Wirral Cancer Centre; The Christie Hospital NHS Foundation; Imperial College Healthcare Trust: St Mary’s Hospital, Hammersmith Hospital, Charing Cross Hospital; University Hospitals of Leicester: The General and the Royal Infirmary; Nottingham University Hospitals: City Hospital.
